# Persistence of *Escherichia coli* in the microbiomes of red Romaine lettuce (*Lactuca sativa* cv. ‘Outredgeous’) and mizuna mustard (*Brassica rapa* var. japonica) - does seed sanitization matter?

**DOI:** 10.1186/s12866-021-02345-5

**Published:** 2021-10-22

**Authors:** Anirudha R. Dixit, Christina L. M. Khodadad, Mary E. Hummerick, Cory J. Spern, LaShelle E. Spencer, Jason A. Fischer, Aaron B. Curry, Jennifer L. Gooden, Gretchen J. Maldonado Vazquez, Raymond M. Wheeler, Gioia D. Massa, Matthew W. Romeyn

**Affiliations:** 1 AMENTUM Services Inc., LASSO, Kennedy Space Center, Merritt Island, FL USA; 2 Southeastern Universities Research Association, LASSO, Kennedy Space Center, Merritt Island, FL USA; 3 NASA UB, Kennedy Space Center, Merritt Island, FL USA

**Keywords:** Microbiome, Phyllosphere, Rhizosphere, Red Romaine lettuce, Mizuna mustard, *E. coli*, ISS, Seed surface sanitization

## Abstract

**Background:**

Seed sanitization via chemical processes removes/reduces microbes from the external surfaces of the seed and thereby could have an impact on the plants’ health or productivity. To determine the impact of seed sanitization on the plants’ microbiome and pathogen persistence, sanitized and unsanitized seeds from two leafy green crops, red Romaine lettuce (*Lactuca sativa* cv. ‘Outredgeous’) and mizuna mustard (*Brassica rapa* var. japonica) were exposed to *Escherichia coli* and grown in controlled environment growth chambers simulating environmental conditions aboard the International Space Station. Plants were harvested at four intervals from 7 days post-germination to maturity. The bacterial communities of leaf and root were investigated using the 16S rRNA sequencing while quantitative polymerase chain reaction (qPCR) and heterotrophic plate counts were used to reveal the persistence of *E. coli*.

**Result:**

*E. coli* was detectable for longer periods of time in plants from sanitized versus unsanitized seeds and was identified in root tissue more frequently than in leaf tissue. 16S rRNA sequencing showed dynamic changes in the abundance of members of the phylum *Proteobacteria, Firmicutes, and Bacteroidetes* in leaf and root samples of both leafy crops. We observed minimal changes in the microbial diversity of lettuce or mizuna leaf tissue with time or between sanitized and unsanitized seeds. Beta-diversity showed that time had more of an influence on all samples versus the *E. coli* treatment.

**Conclusion:**

Our results indicated that the seed surface sanitization, a current requirement for sending seeds to space, could influence the microbiome. Insight into the changes in the crop microbiomes could lead to healthier plants and safer food supplementation.

**Supplementary Information:**

The online version contains supplementary material available at 10.1186/s12866-021-02345-5.

## Introduction

Microbial interactions on, in, and around the seeds can have profound effects on plant growth, development, and productivity. These interactions can be casual or intimate in nature, but ultimately, they all contribute in varying degrees to an ever-evolving microbiome. Plant microbiomes have been investigated over decades and new data continue to reveal how microbiomes play an important role in plant success. Many eubacteria and fungi have been found to have a symbiotic relationship with plants and other microorganisms. The source of the plant microbiome on adult plants is provided by the seed via vertical transmission to leaf, root, flowers or fruit [1], as well as the surrounding environment.

Plant-pathogens can be associated with the plant microbiome, some broad ranging, infecting multiple plant species, and others being host-specific. Damage to the hosts can be significant, causing economic and yield impacts [2]. Examples such as *Pseudomonas, Ralstonia,* and *Agrobacterium* pathovars can cause significant damage in crop plants and all have economic impacts [2]. However, studies of these genera have provided scientific breakthroughs on plant diseases. Plant tissue also has the potential to carry non-plant pathogen associations.

In recent decades, leafy greens have been associated with large outbreaks of food-borne illness including enterohemorrhagic *Escherichia coli* (*E. coli)* infections [3, 4]. Enteric human pathogens on produce are introduced through a variety of sources and production steps from farm to market. Studies have shown that *E. coli* can colonize and persist in low numbers on plant surfaces [5–7] and that colonization depends on a variety of abiotic and biotic factors [8, 9]. The impact of indigenous plant microbial communities on the persistence of human pathogens on plant surfaces and the inverse is unclear, and the subject of recent scientific investigations [10–12]. It has been shown that native microflora may play a role in pathogen suppression on fruits and vegetables, leading to studies on the development of biological control inoculants to inhibit the proliferation of human pathogens on produce [13–15].

National Aeronautics and Space Administration (NASA) has been growing plants on the International Space Station (ISS) to supplement the packaged astronaut diet since 2014, and food safety is critical. Plant tissue and rooting pillows returned from ISS have been compared to parallel ground controls to evaluate the presence of microbes from a food safety point of view [16]. These initial studies utilized the 16S rRNA gene and the interspatial transcribed sequence (ITS) gene to identify the community players. However, the role of these microbes as members of the microbiome remains a mystery. The degree to which crop cultivation in closed, isolated environments like the ISS may have on the development of the plant microbiome and consequent human pathogen proliferation is not understood.

The aim of this study was to investigate the microbiomes of the plant’s phyllosphere, rhizosphere, and respective endosphere when challenged with *E. coli,* strain ATCC 11775 as a surrogate for the pathogenic *E. coli* O157:H7 [17, 18] and grown under simulated ISS environmental conditions. Seeds from two leafy green crops that have previously grown on the ISS, specifically red Romaine lettuce, *Lactuca sativa* cv. ‘Outredgeous,’ and mizuna mustard, *Brassica rapa* var. *japonica,* were exposed to *E. coli.* During a time-course study, using bacterial plate counts, quantitative polymerase chain reaction (qPCR), and 16S rRNA amplicon sequencing, survival of the pathogen and characterization of the plant community were traced from germinated seeds to the mature plants. Recent advances in sequencing technologies and parallel omics methods continue to increase our knowledge of community characterization as well as community function and understanding the microbial communities of the plant-associated microbiome separated into above-ground (phyllosphere) and below-ground (rhizosphere) sections is vital to understanding plant response, impact on plant health, and food safety.

## Results

### Bacterial population dynamics

**Fig. 1:**
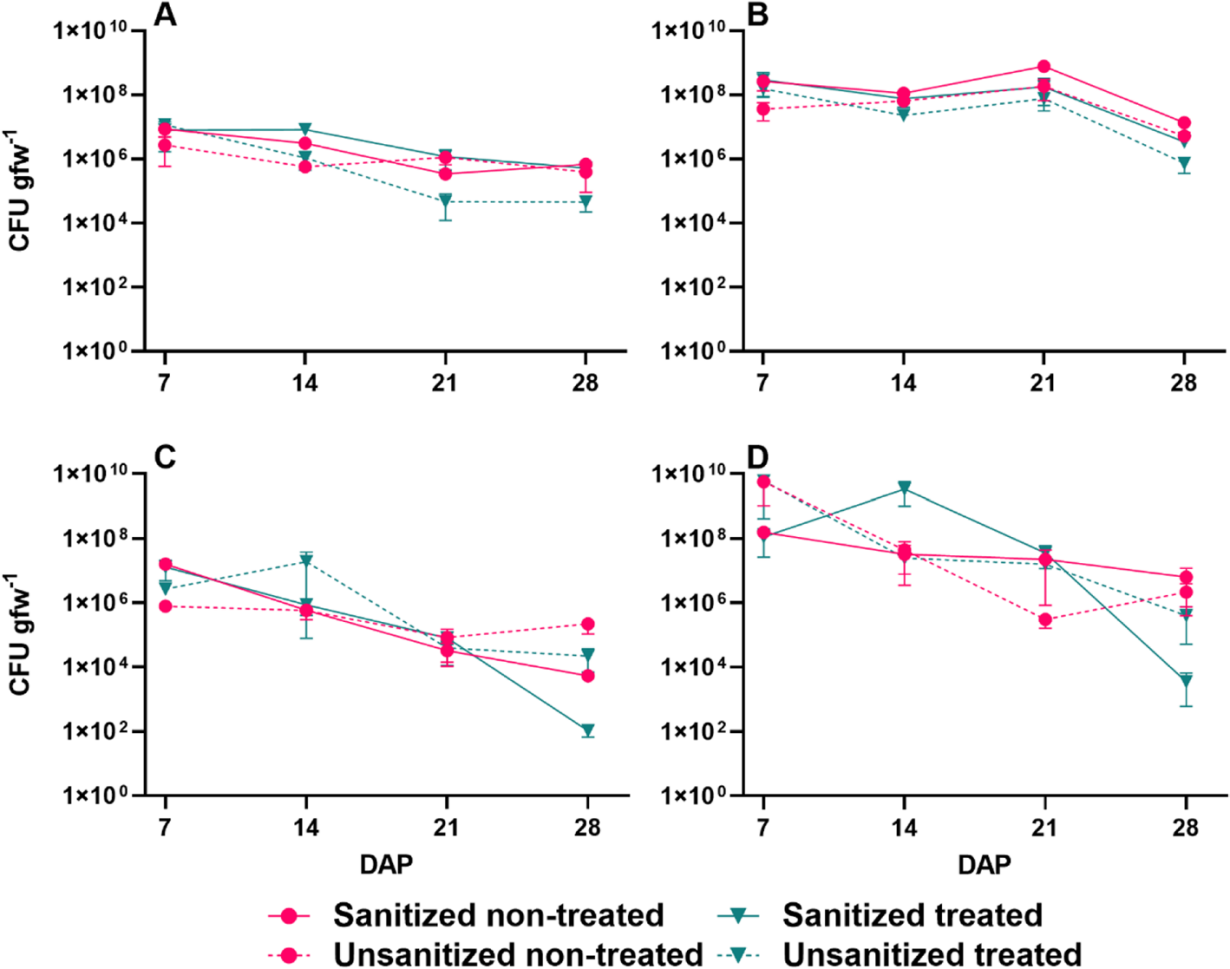
Aerobic plate counts per gram fresh weight on red Romaine lettuce and mizuna mustard. Plants were grown from either sanitized or unsanitized seeds that were either treated or non-treated with *E.coli*. Lettuce leaves (**A**) and roots (**B**); mizuna mustard leaves (**C**) and roots (**D**). Each point represents the average and bars represent SEM (*n* = 3)

Bacterial abundance of the phyllosphere of lettuce and mizuna as indicated by aerobic plate counts per gram fresh weight (APC gfw^− 1^) on TSA (Trypticase Soy Agar) generally declined through the growth period from day 7 to day 28. On the lettuce leaves, the average APCs at day 7 were in the range of 2.69 × 10^6^–1.15 × 10^7^ colony forming units (CFU) gfw^− 1^ for plants from sanitized and unsanitized seeds, both treated and non-treated with *E. coli.* Bacterial numbers declined approximately 1 order of magnitude on leaf samples from all the treatments except for the unsanitized seeds treated with *E. coli.* The counts on these samples were 1.15 × 10^7^ CFU gfw^− 1^ at day 7 declining to significantly lower levels (*P* = 0.03) when compared to the leaf tissue from sanitized seeds treated with *E. coli* reaching 4.62 X 10^4^ CFU gfw^− 1^ at 21 days and leveling off through day 28 (Fig. 1A).

Similar to the bacterial counts from the lettuce, average counts for mizuna leaf samples at day 7 ranged from log 7.9 × 10^5^ CFU gfw^− 1^ on the non-treated seeds that were not sanitized to the highest level of log 1.31 × 10^7^ CFU gfw^− 1^ on the non-treated sanitized seeds. This difference was significant (*P* = 0.0095) but did not persist throughout the 28-day growth period. As was seen with lettuce counts, the APC gfw^− 1^ declined over the 28-day period, but the rate varied with each treatment. Leaves from unsanitized, *E. coli* non-treated seeds had the highest CFU counts, 2.23 × 10^5^ after 28 days followed by the plants from seeds that were not sanitized but treated with *E. coli* at 2.2 × 10^4^ CFU gfw^− 1^. The lowest counts for the day 28 leaf samples were from seeds that were sanitized and treated with *E. coli* with average counts of 1.05 × 10^2^ CFU gfw^− 1^, significantly lower (*P* = 0.03) than the leaves from the unsanitized *E.coli* treated seeds (Fig. 1C).

Bacterial counts from roots of both leafy greens were higher than leaves, ranging from 3.62 × 10^7^ to 2.88 × 10^8^ CFU gfw^− 1^ in lettuce and 1.14 × 10^8^ to 6.07 × 10^9^ CFU gfw^− 1^ in mizuna at day 7. As with the leaf tissue, a decline in abundance was observed over time. There was no significant difference between the counts on lettuce root tissue with different seed treatments (Fig. 1B). After 28 days of growth, the counts on mizuna roots from sanitized *E.coli* treated seeds was 3.62 × 10^3^ CFU gfw^− 1^, a significantly lower number than those from roots from all other seed treatments (Fig. 1D).

### *E. coli* enumeration on leaf and root

*E. coli* on treated sanitized and unsanitized seeds and plant tissues from treated sanitized and unsanitized seeds was enumerated using culture-independent quantitative PCR. *E. coli* cells per seed from each treatment and seed type after incubation in TSB (Trypticase Soy Broth) (T = 0) were 5.3 × 10^4^ for sanitized lettuce, 8.2 × 10^4^ for unsanitized lettuce seeds, 4.3 × 10^4^ for sanitized mizuna and 2.5 × 10^4^ for the unsanitized mizuna.

**Fig. 2:**
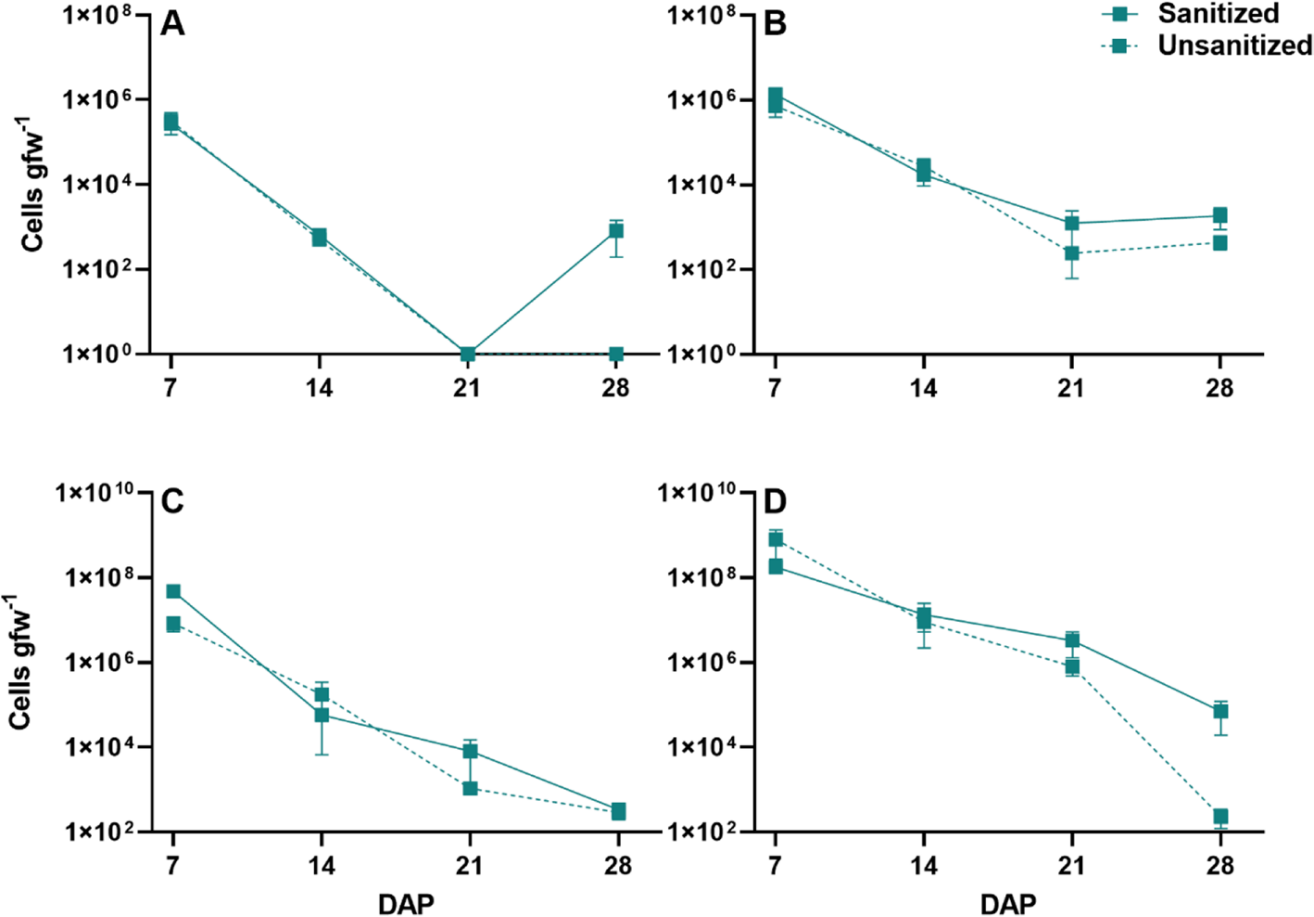
*E. coli* counts per gram fresh weight for red Romaine lettuce and mizuna mustard as determined by qPCR. Plants were grown from either sanitized or unsanitized seeds that were treated with *E. coli*. Lettuce leaf (**A**) and roots (**B**); mizuna mustard leaves (**C**) and roots (**D**). Each point represents the average and bars represent SEM (*n* = 3)

This assay demonstrated a decline in *E. coli* abundance over the 28-day growth period in all plant tissues tested. In lettuce leaves, at days 7–21 there was no significant difference in *E. coli* cell counts between the seed treatments. Over the 28-day growth period, cell counts from the lettuce leaves from sanitized seeds declined approximately 3 orders of magnitude from 3.2 × 10^5^ at day 7 to 8.11 × 10^2^ cells gfw^− 1^ at harvest (day 28), whereas *E. coli* levels for leaves from the unsanitized seeds remained undetectable after 21 days. At day 21, *E. coli* was not detected in either treatment, but at day 28 *E. coli* was detected in the sanitized seed treatment only, indicating a significant difference at day 28 between lettuce leaves from sanitized and unsanitized seeds (Fig. 2A).

Enumeration of *E. coli* on lettuce roots from sanitized and unsanitized seeds indicated no significant difference between the treatments over time. Roots from sanitized and unsanitized seeds, showed a decline in average cell counts gfw^− 1^ from 1.36 × 10^6^ to 1.86 × 10^3^ and 7.28 × 10^5^ to 4.39 × 10^2^ respectively. Hence, *E. coli* was detected in the roots after 28 days of growth at harvest (Fig. 2B). Similar to lettuce leaves, *E. coli* from mizuna leaves declined over the course of 28 days of growth from 4.77 × 10^7^ cells gfw^− 1^ to 2.90 × 10^2^ cells gfw^− 1^ in the leaves from sanitized seeds and 8.34 × 10^6^ cells gfw^− 1^ to 2.9 × 10^2^ cells gfw^− 1^ in the leaves from unsanitized seeds. There were no differences in the qPCR data between seed sanitization treatments through the time course however, *E. coli* was detected in both mizuna leaf samples at day 28 (Fig. 2C). Mizuna roots from sanitized and unsanitized seeds showed no significant difference in *E. coli* counts between the treatments over time. For the roots from sanitized and unsanitized seeds, the average cell counts gfw^− 1^ declined from 1.78 × 10^8^ to 7.05 × 10^4^ and 7.96 × 10^8^ to 2.3 × 10^2^ respectively. As in the lettuce roots, *E. coli* was detected after 28 days of growth at harvest (Fig. 2D).

### Characterization and abundance of bacterial communities

To understand the combined effects of seed sanitization and *E. coli* treatment on plant-associated microbial communities, a 16S rRNA amplicon sequencing was completed comparing leaf and root microbial communities separately. The sequencing data contained, after removing chimeric and unassigned sequences, a total of 16,518,574 and 17,991,042 (leaf and root, forward and reverse) reads for lettuce and mizuna, respectively. After removal of chloroplast and mitochondrial taxonomic assignments, there were 3,713,493 and 3,691,028 high-quality sequences for lettuce and mizuna, respectively.

**Fig. 3:**
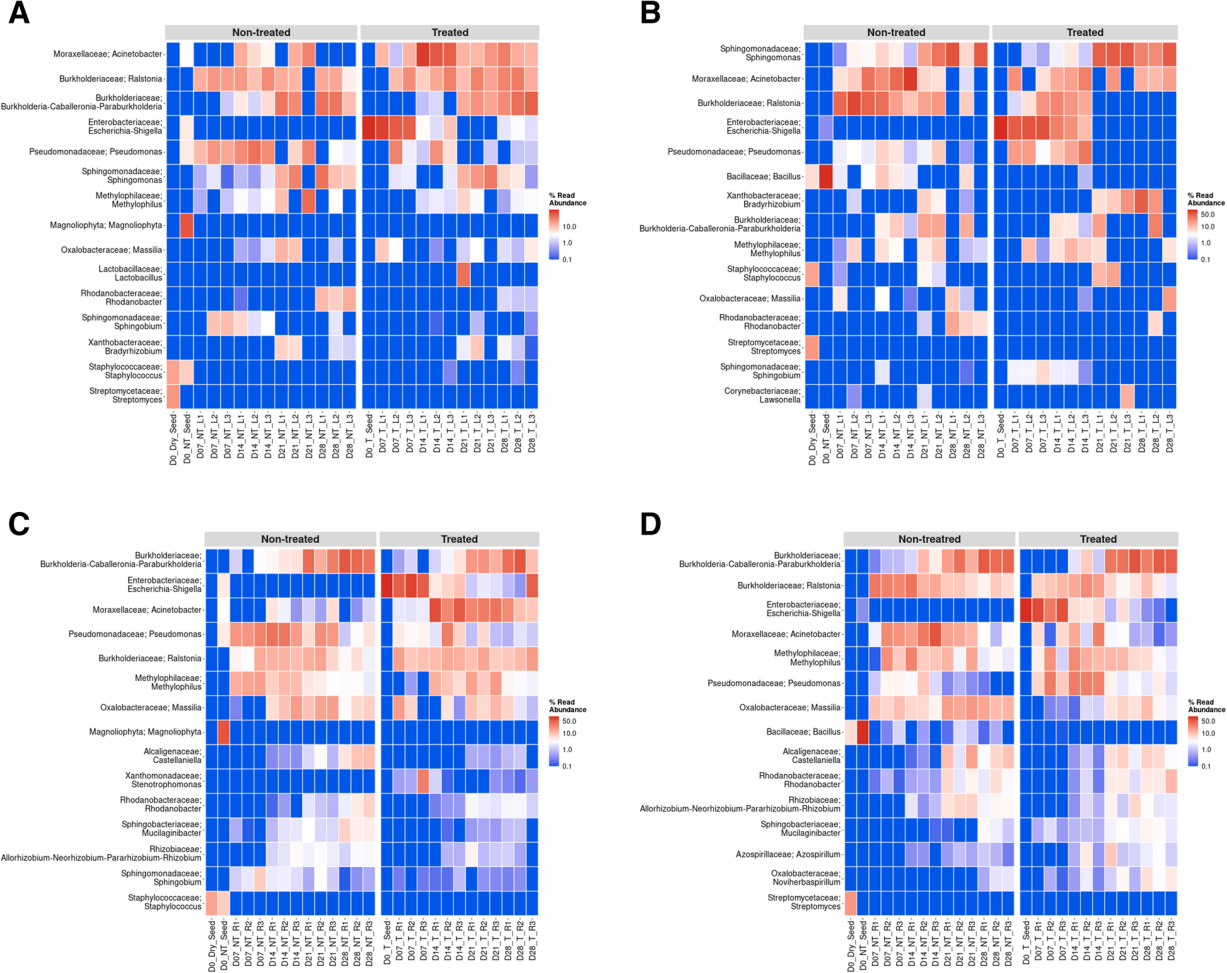
Heatmaps showing the 15 most abundant genera as percent read abundance for bacterial populations in red Romaine lettuce. Columns represent leaf (L) or root (R) samples, from sanitized (**A, C**) or unsanitized (**B, D**) seeds, respectively, over time. Samples were either treated (T) or non-treated (NT) with *E. coli.* Numbers on X-axis preceded by the letter D represent days after planting (DAP)

**Fig. 4:**
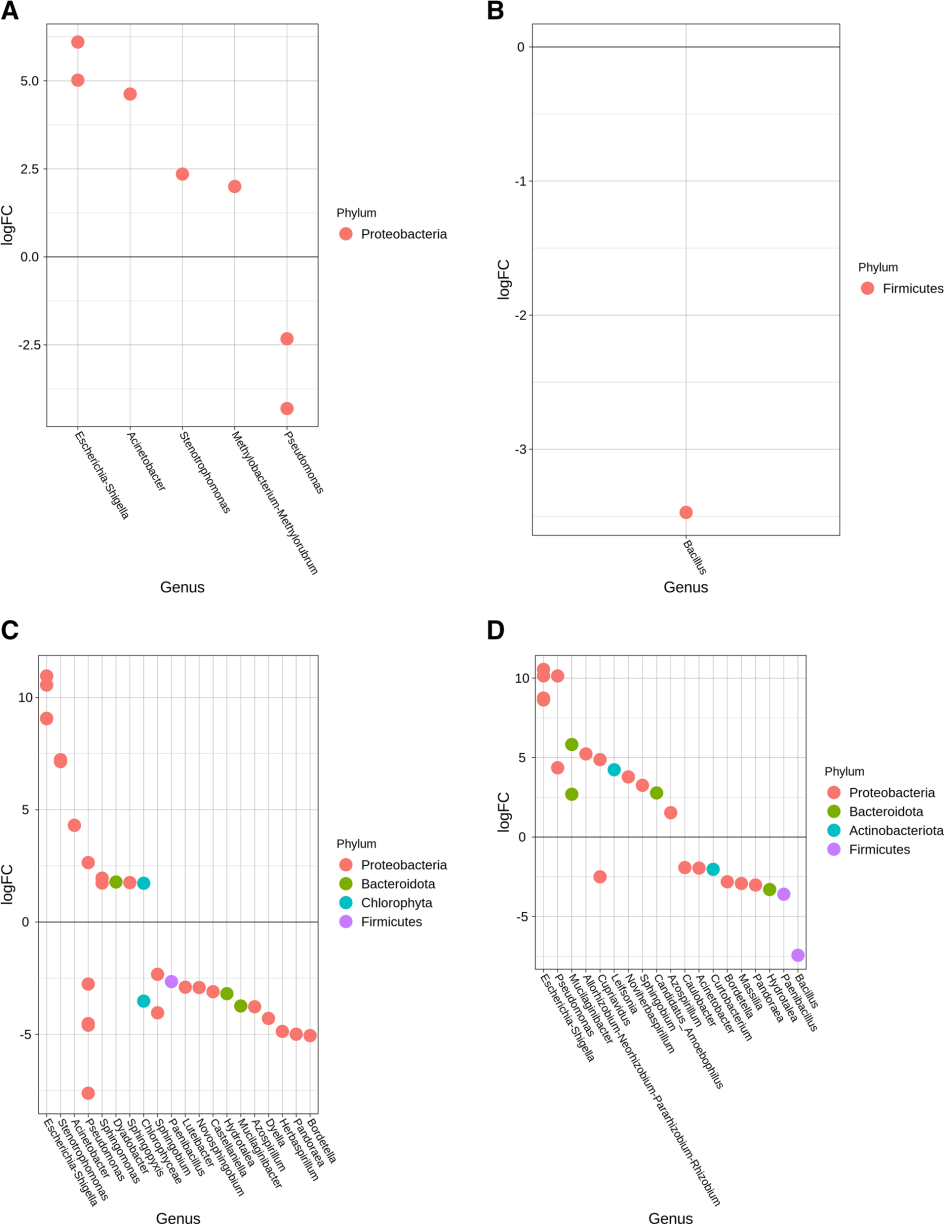
Differential abundance plots showing bacterial genera that are significantly different in red Romaine lettuce (**A-D**). Panels (**A**) and (**B**) represent leaves, (**C**) and (**D**) represent roots from either sanitized or unsanitized seeds, respectively. Colors indicate the phylum each genus belongs to with significant differential abundance determined at *P* < 0.05

Comparison of the relative abundances of the bacterial communities between lettuce leaves from sanitized and unsanitized seeds showed differences in the way *E. coli* persisted in treated samples. Between days 7 and 14, the average relative abundance for *E. coli* dropped from 40 to 15%, specifically, in the case of day 14, there was a notable change (~ 10%) in *E. coli* relative abundance between leaves from unsanitized and sanitized seeds. Genera such as *Pseudomonas*, *Ralstonia,* and *Acinetobacter* also showed dynamic changes in relative abundance during days 7 and 14. *Sphingomonas, Burkholderia,* and *Bradyrhizobium* exhibited dynamic changes in their relative abundances during days 21 and 28, when *E. coli* began to decline (Fig. 3A & B). The differential abundance analysis showed the above-mentioned genera to be common across all time points except for the presence of the genus *Bacillus,* whose abundance increased by more than 2-fold in unsanitized samples (Fig. 4A & B).

In the case of roots from sanitized and unsanitized seeds, 89% of the genera represented were common between both sample types, across all time points with the exception of *Sphingobium* and *Stenotrophomonas*, which were present exclusively in the plants generated from sanitized seed whereas the genera *Bacillus* and Azospirillum were present only in the unsanitized samples (Fig. 3C & D). *E. coli* relative abundance followed a similar pattern for roots in plants from sanitized and unsanitized seeds, starting with the highest abundance at day 7 and gradually decreasing to less than 1% by day 28. Similar to leaves, genera such as *Burkholderia-Caballeronia-Paraburkholderia*, *Acinetobacter*, *Ralstonia*, *Methylophilus*, *Pseudomonas,* and *Massilia* exhibited dynamic changes in relative abundance, sometimes showing an inverse relationship to *E. coli* (Fig. 3C & D). Between lettuce roots from sanitized and unsanitized seeds, a dominance of phyla *Proteobacteria*, *Firmicutes,* and *Bacteroidetes* was observed. In particular, genera such as *Pantoea*, *Pseudomonas,* and *Sphingobium* showed an increased abundance whereas *Sphingomonas*, *Acinetobacter*, *Stenotrophomonas,* and *Escherichia-Shigella* showed an overall decrease in abundance throughout the study (Fig. 4C and D).

**Fig. 5:**
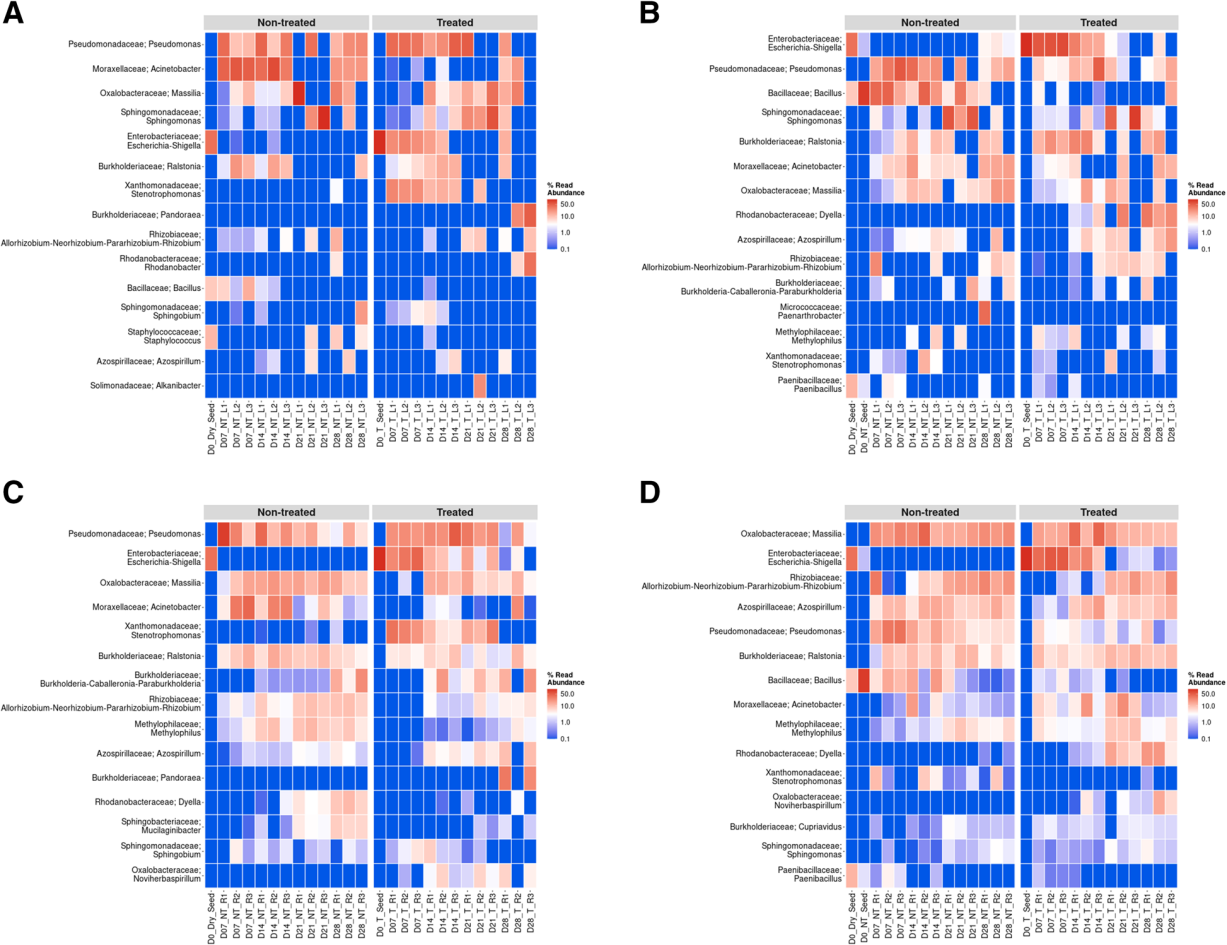
Heatmaps showing the 15 most abundant genera as percent read abundance for bacterial populations in mizuna mustard. Columns represent leaf (L) or root (R) samples, from sanitized (**A, C**) or unsanitized (**B, D**) seeds, respectively, over time. Samples were either treated (T) or non-treated (NT) with *E. coli.* Numbers on X-axis preceded by the letter D represent days after planting (DAP)

**Fig. 6:**
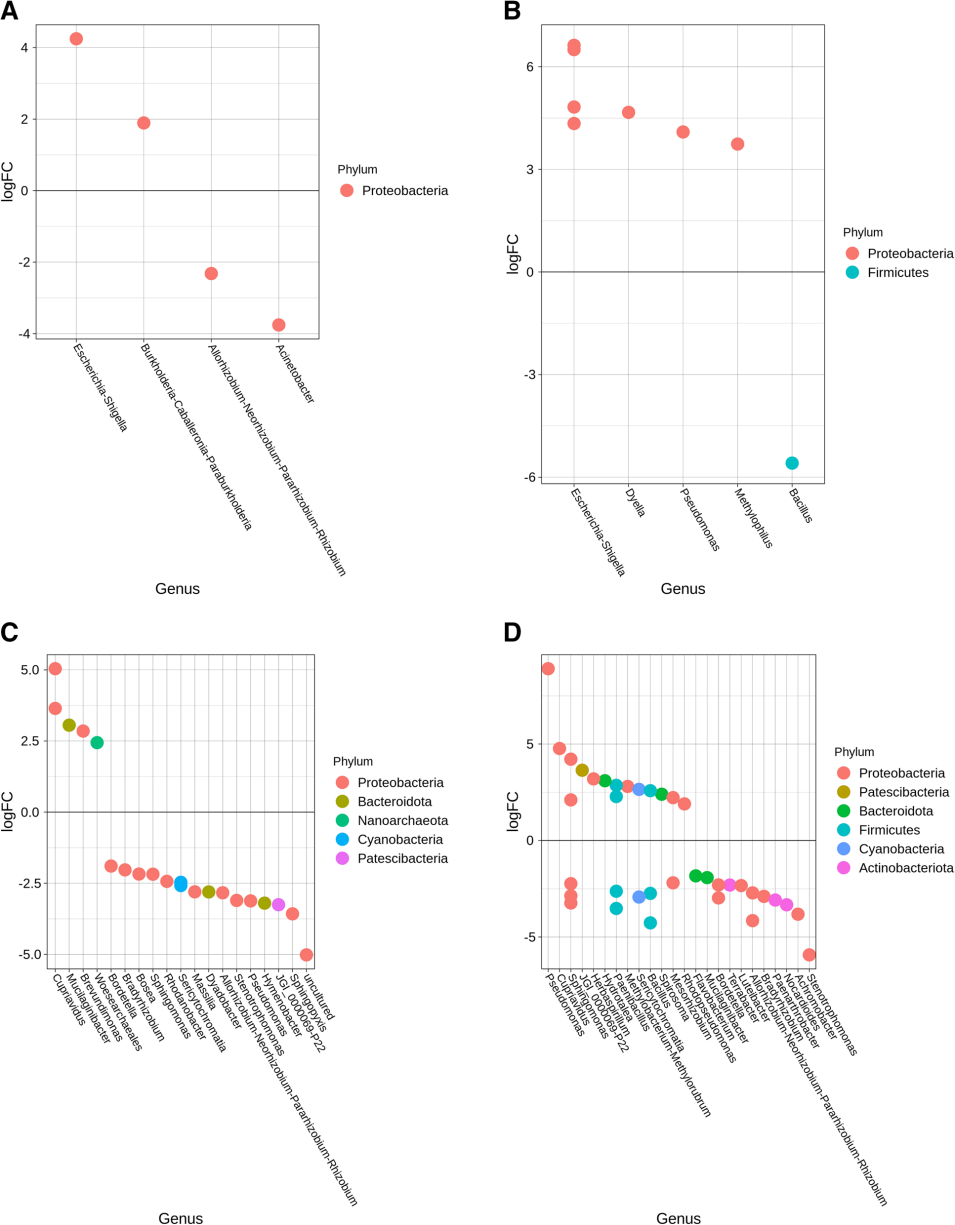
Differential abundance plots showing bacterial genera that are significantly different in mizuna mustard (**A-D**). Panels (**A**) and (**B**) represent leaves, (**C**) and (**D**) represent roots from either sanitized or unsanitized seeds, respectively. Colors indicate the phylum each genus belongs to with significant differential abundance determined at *P* < 0.05

Comparing mizuna leaves from sanitized and unsanitized seeds, more than 78% of the genera were common between the two types of samples, with the exception of *Dyella, and Rhodanobacter* being exclusively present in unsanitized samples with *Pandoraea* and *Sphingobium* present in sanitized samples. Leaves from sanitized and unsanitized seeds treated with *E. coli* did show a gradual decrease in *E. coli* abundance from an average of 44% at day 7 to less than 1% at day 28. At the same time, other genera such as *Pseudomonas*, *Acinetobacter*, *Ralstonia,* and *Stenotrophomonas* exhibited dynamic shifts in their relative abundance at days 7 and 14, whereas *Sphingomonas* showed a gradual increase in abundance until day 21 (Fig. 5A & B). Differential abundance analysis of mizuna leaves from sanitized and unsanitized seeds showed an increased abundance for genera *Acinetobacter* and *Bacillus,* while *Pseudomonas* and *Escherichia-Shigella* showed a decrease in their abundance across all time points studied (Fig. 6A & B).

Mizuna roots on the other hand had approximately 73% of the taxa commonly represented between samples from sanitized and unsanitized seeds with each of them retaining 13% taxa exclusively. Similar to leaves, comparison of the roots from sanitized and unsanitized seeds showed a gradual decrease in relative abundance of *E. coli* from an average of 44% at day 7 to less the 1% at day 28. At the same time, some of the most abundant genera such as *Massilia, Allorhizobium-Neorhizobium-Pararhizobium-Rhizobium, Azospirillum, Pseudomonas,* and* Acinetobacter *showed dynamic changes in their relative abundance especially in day 7 and day 14 (Fig. 5C & D). These same genera were also found to be differentially abundant across all the time points studied representing some of the dominant phyla such as *Proteobacteria, Firmicutes, Bacteroidetes,* and *Actinobacteria* (Fig. 6C & D)*.*

### Diversity within bacterial communities

Alpha and beta diversity provided overall community characterization of abundance and distribution within and between samples over time. Alpha diversity, determined by the Shannon Index, revealed that diversity for both sanitized and unsanitized seed-generated plant tissues generally fell between 1.0 and 4.0 for both red Romaine lettuce and mizuna mustard, which were within normal range for environmental samples [17–19]. There was no significant difference in diversity between time points for all lettuce *E. coli* treated and non-treated samples for both sanitized and unsanitized seeds with the exception of between day 7 and day 28 in sanitized *E. coli* treated leaf tissue (*P* = 0.033) and unsanitized *E. coli* non-treated lettuce root tissue (*P* = 0.034). A detailed investigation into the lettuce samples indicated that there was an increase in the number of genera with time and nearly three times more genera in the day 28 replicates than in the day 7 replicates (Fig. S1).

The comparison between time points in each treatment type for mizuna mustard showed no significant differences in the overall alpha diversity values in leaf tissue (*P* > 0.05) with two exceptions in mizuna root tissue. A closer inspection of the alpha diversity of mizuna roots indicated that there was a similar increase in diversity with time and the number of species correspondingly increased as well. In the mizuna root of unsanitized, non-treated samples, the number of genera nearly tripled while in the unsanitized *E. coli* treated samples the number of genera only doubled between days 7 and 28 (Fig. S2).

**Fig. 7:**
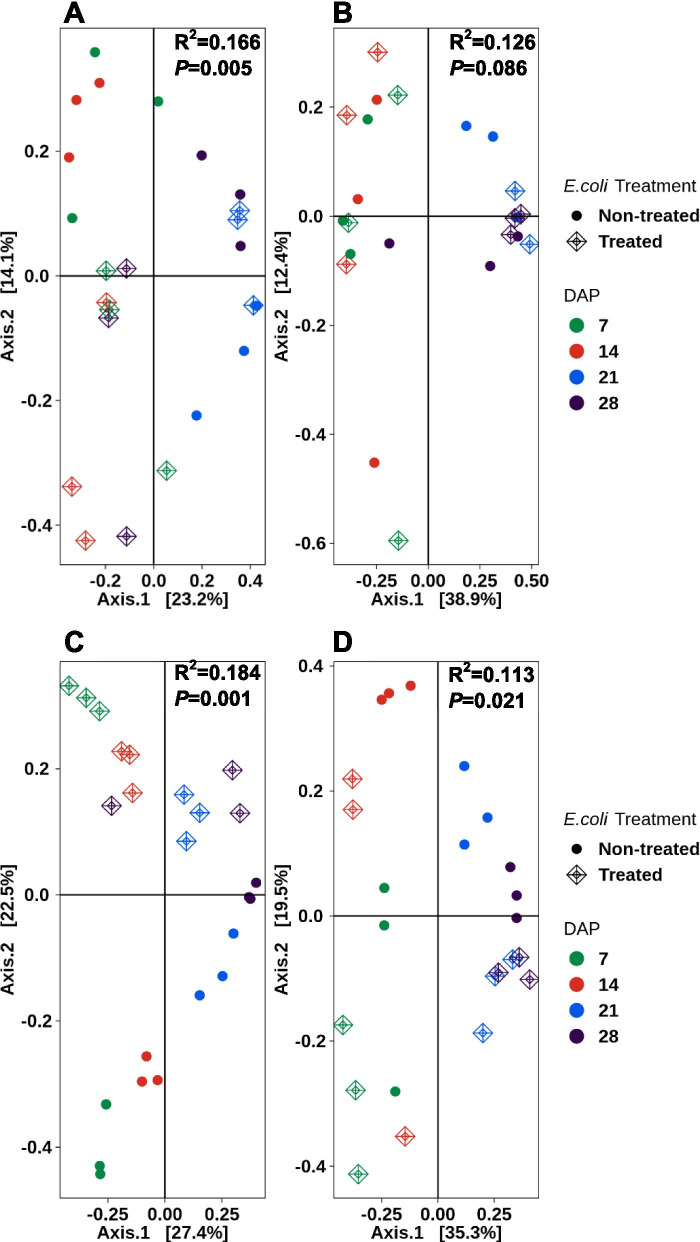
PCoA plots for beta diversity (Bray-Curtis dissimilarity) in red Romaine lettuce, with time (DAP) versus *E. coli* treatment. Increasing distance in two-dimensional space represents increasingly dissimilar community structures. Leaves from sanitized (**A**) and unsanitized (**B**) seeds. Roots from sanitized (**C**) and unsanitized (**D**) seeds. The R^2^ value represents percent variance that can be explained by the specified groups (DAP + *E. coli* treatment) as calculated using adonis, a multivariate PERMANOVA test implementation in QIIME2

Beta diversity determines the differentiation between samples. The Bray-Curtis statistic (R^2^) was used to calculate the dissimilarity between *E. coli*-treated, and non-treated samples, along the time course. A higher dissimilarity exists at values closer to 1.0 and a similarity exists closer to zero. We determined that beta diversity in lettuce leaves grown from the sanitized seeds presented a significant difference (*P* < 0.05) (Fig. 7A). The differences explained in two axes (time and *E. coli* exposure) were low, at 37.3% and there appeared to be a significant variation in the sample distribution (*P* = 0.005). Lettuce leaf tissue from the sanitized, non-treated seed generated samples clustered more closely for days 7, 14 and 28, indicating microbiomes that are more similar. Day 21 for this treatment diverged from this outcome and the three replicates were more dissimilar. The lettuce leaf tissue generated from sanitized, *E. coli*-treated seeds did not cluster indicating the samples were not similar to each other. The diversity comparisons indicated that time (R^2^ = 0.301) had a greater effect than the *E. coli* treatment (R^2^ = 0.093). Both were significant (*P* = 0.001) (Table 1). The leaf tissue generated from unsanitized seeds presented a similar clustering pattern; however, this pattern was translocated to a different quadrant and distributed across axes (Fig. 7B). The data from these two axes represented approximately 51% of the influence of the two parameters with cluster separation of days 7 and 14 from days 21 and 28. Days 21 and 28 clustered more tightly toward the right quadrants inferring that time may have a greater influence on the microbiome. The variation in the unsanitized seed-generated leaf tissue was not significant (*P* = 0.086) (Fig.7B). In addition, there was no clustering between the *E. coli* treated and non-treated tissue indicating dissimilarity between these samples. However, when time and *E. coli* treatment were considered independently, time could explain about 40% of the variation, and the *E. coli* treatment explained only 7.6% (Table 1). Both were significant (*P* = 0.001 and *P* = 0.02, respectively).

**Table 1:** Adonis test of factors (TimePoint (DAP), *E.coli* treatment and combined effects) explaining distance variation in Bray-Curtis dissimilarity for lettuce and mizuna

	TimePoint		*E. coli* Treatment		TimePoint + *E. coli* Treatment	
	R^2^	P	R^2^	P	R^2^	P
**Lettuce**						
Leaves from sanitized seeds	0.301	0.001	0.093	0.001	0.166	0.005
Leaves from unsanitized seeds	0.401	0.001	0.076	0.020	0.126	0.086
Roots from sanitized seeds	0.348	0.001	0.198	0.001	0.184	0.001
Roots from unsanitized seeds	0.462	0.001	0.126	0.001	0.113	0.021
**Mizuna Mustard**						
Leaves from sanitized seeds	0.426	0.001	0.064	0.012	0.126	0.043
Leaves from unsanitized seeds	0.422	0.001	0.095	0.001	0.138	0.004
Roots from sanitized seeds	0.293	0.001	0.269	0.001	0.175	0.001
Roots from unsanitized seeds	0.293	0.001	0.160	0.001	0.218	0.001

Figure 7C (sanitized) and 7D (unsanitized) illustrate the beta diversity or the lettuce root tissue. The PCoA in Fig. 7C and D accounted for 50 and 55% of the variation, respectively. The beta diversity within root tissues indicates a near reversal of the information gleaned from the lettuce leaf tissue. The replicates from the sanitized seed-generated root tissue, which had been exposed to *E. coli*, clustered tightly with the exception of day 28 (Fig. 7C). The replicates from each of the non-treated tissues also grouped tightly together, but were separated by time, with day 7 and 14 being influenced negatively by both axes, and day 28 influenced positively by both axes. Overall, time could explain approximately 35% of the variation, while the *E. coli* treatment could account for 20% with both being significant (*P* = 0.001) (Table 1). The diversity within root tissues generated from unsanitized seeds showed less clustering, indicating more dissimilarity than the sanitized seed-generated root tissue with day 7 root tissue presenting the most dissimilarity (Fig. 7D). Days 7 and 14 *E. coli* treated plants did not show any clustering, and were therefore dissimilar, while days 21 and 28 grouped more closely to each other but differently from days 7 and 14 (Fig. 7D). This indicates a similarity to each other but more variation between time points, which could account for 46.2% of the variation while the *E. coli* treatment accounted for 13%. Both were significant (*P* = 0.001) (Table 1).

**Fig. 8:**
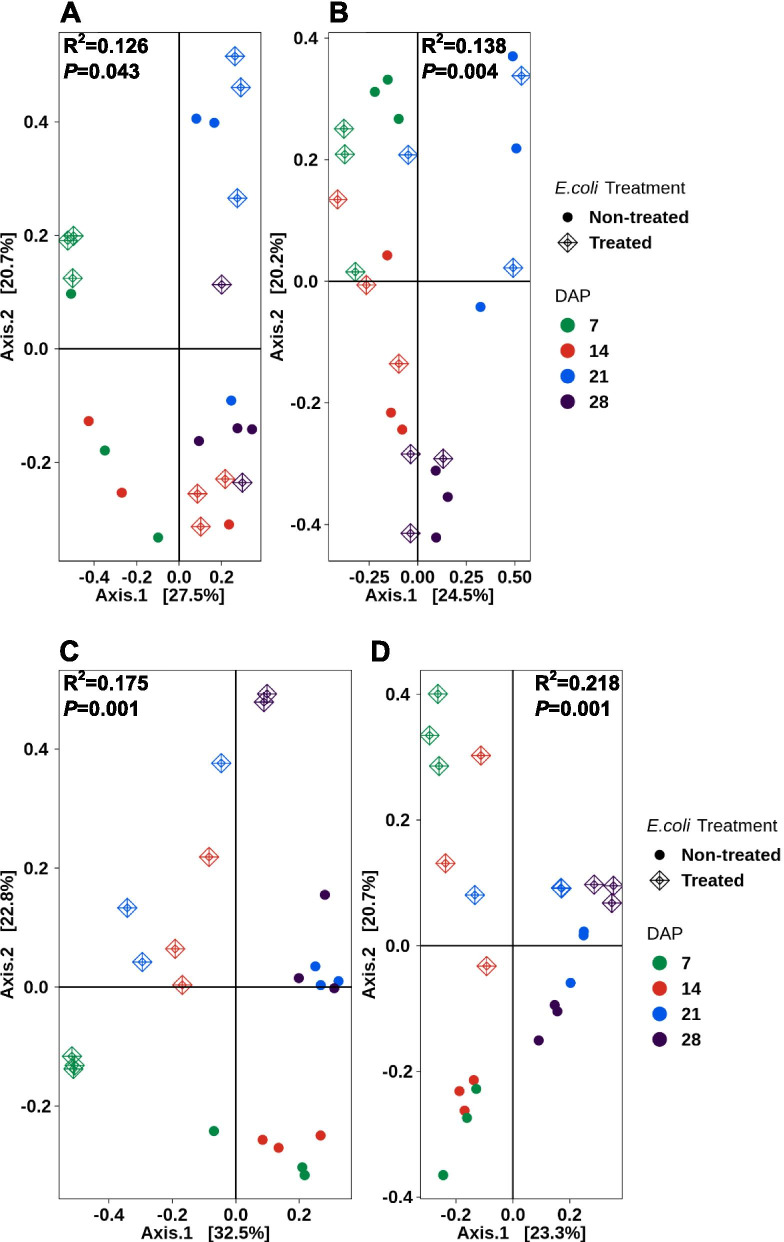
PCoA plots for beta diversity (Bray-Curtis dissimilarity) in mizuna mustard, with time (DAP) versus *E. coli* treatment. Increasing distance in two-dimensional space represents increasingly dissimilar community structures. Leaves from sanitized (**A**) and unsanitized (**B**) seeds. Roots from sanitized (**C**) and unsanitized (**D**) seeds. The R^2^ value represents percent variance that can be explained by the specified groups (DAP + *E. coli* treatment) as calculated using adonis, a multivariate PERMANOVA test implementation in QIIME2

Beta diversity in the mizuna leaf tissue from sanitized seeds indicated little similarity between the *E. coli*-treated and non-treated leaves. The PCoA for sanitized and unsanitized seed generated leaf tissue explained 48.2 and 44.7% of the variation, respectively. (Fig. 8A and B). Mizuna samples from sanitized, *E. coli-*treated seeds showed some grouping between samples (i.e. day 7 and 14) however, only 12% of the dissimilarity could be explained by both parameters combined. Time alone could explain approximately 42%, but the *E. coli* treatment explained only 6% (Table 1). The non-treated samples showed increased variation with the exception of day 28. There was some visible clustering between *E. coli* treated and non-treated samples but there was a clear separation of those samples with time. Similar trends were seen with the unsanitized seed-grown mizuna leaf tissue. There was little clustering between mizuna leaf tissue from *E. coli*-treated and non-treated seeds with the exception of day 28 indicating a similarity in the microbiomes (Fig. 8B). Approximately 42% of the variation was due to time, while the *E. coli* treatment provided only 9%. Together, the influence dropped drastically to 13.8%. All were a significant influence (*P* < 0.05). The mizuna root showed a different clustering pattern from the leaf tissue. The combined axes for the sanitized seed-grown root tissue explains 55.3% of the variation (*P* = 0.001), while the unsanitized seed-grown root tissue explains 44% of the variation (*P* = 0.001) (Fig. 8C & D). There was some grouping among the replicates for both treated and untreated samples with some similarity between day 7 and 14, and between day 21 and 28. However, there was little clustering between the *E. coli*-treated and non-treated samples. This indicated a dissimilarity in the microbiomes generated and the R^2^ values of 0.175 and 0.218 support this outcome (Table 1). In the case of mizuna root tissue, in both sanitized and unsanitized seed treatments, time played a greater role (29%) in the variation than did the *E. coli* exposure (26 and 16% respectively). All were significant (*P* = 0.001).

## Discussion

This study examined the dynamics of the culturable, indigenous, aerobic, bacterial population and the persistence of *E. coli* introduced on sanitized and unsanitized seeds in the phyllosphere and rhizosphere of mizuna and lettuce. To minimize the introduction of potential plant and human pathogens on food crops grown on ISS, seeds are routinely sanitized [20] consequently eliminating most of the seed microbial epiphytes. Microbial abundance on seeds is significant with culturable microbial counts ranging from 10^3^ to 10^7^ depending on the plant [21–23], therefore surface sanitizing in an effort to eliminate potential pathogen transmission to the plant initiates a perturbation to the seed microbiome.

The influence of the seed microbiome on the development of the phyllosphere and rhizosphere microbial communities has been studied in a variety of plants [24–26]. Links et al. [23] characterized the bacterial and fungal populations on the surface of wheat and canola seeds and identified the bacterium *Pantoea agglomerans* with antagonistic properties toward a fungal isolate on both seed types. The effect of removing seed surface microbes by sanitization on the development and function of succeeding plant microbial communities and pathogen proliferation, to our knowledge, has not been studied. In the present study, both sanitized and unsanitized seeds of lettuce and mizuna were inoculated with *E. coli* to determine the impact of the seed surface sanitizing treatment, if any, on the growth and persistence of the pathogen on leaves and roots.

Aerobic bacterial counts on the phyllosphere of a variety of leafy greens have been reported in the range of 10^1^ to 10^8^ CFU g^− 1^ [9, 27], which is consistent with our results for red Romaine lettuce and mizuna mustard through the 28-day growth period. At day 28, red Romaine lettuce from unsanitized, *E. coli* treated seeds had a significantly lower CFU count gfw^− 1^ and *E. coli* was not detected in the 21 and 28-day samples. Seeds that had been sanitized and treated with *E. coli* maintained a higher CFU count gfw^− 1^ throughout the growth period, and *E. coli* was detected in the 28-day samples. This trend suggests a seed sanitization effect on the abundance of bacteria and *E. coli* persistence on the red Romaine lettuce leaves, possibly due to the sustained presence of competing bacterial species on the plants from unsanitized seeds. Seed inoculation of native plant rhizosphere or antagonistic bacteria has proven to be an effective biocontrol to prevent the proliferation of pathogens in seed sprouts [28]. This trend was not evident in the mizuna mustard leaf bacterial counts with no difference between the treatments through 21 days of growth followed by a significant decrease in APC on the leaves from sanitized, *E. coli* treated seeds at day 28 and no significant difference in the *E. coli* counts. *E. coli* was detected after 28 days in all the mizuna leaf and root samples, although steadily declining through the 28-day growth period.

Diversity in these bacterial communities may play a role or serve as indicators of plant health. Phyllosphere bacteria play important roles as they provide a resource for nutrient cycling [29, 30] or prevent pathogens from colonizing leaf surfaces [31, 32]. Microbial endophytes in healthy plants would have a positive influence on the host but could also play a role in microbe-microbe interactions [33, 34]. Early colonization would set up or establish varying community compositions in the phyllosphere [35]. By altering the microbial community of the seed, it is suspected that the microbiomes would vary, with a potential to affect plant health.

Through 16S rRNA gene amplicon sequencing, we identified *Proteobacteria, Actinobacteria, Bacteroidetes, and Firmicutes* as the most prevalent phyla in red Romaine lettuce leaves and roots, irrespective of seed sanitization status. This is consistent with previous studies of bacterial communities on leafy greens [8, 10, 11, 14, 36, 37]. Our results showed that, in red Romaine lettuce roots, the relative abundance of *Actinobacteria* and *Bacteroidetes* gradually increased through different developmental stages, which was consistent with observations by Chaparro et al. [38].

In the case of mizuna mustard, we observed *Proteobacteria*, *Firmicutes*, *Actinobacteria*, *Cyanobacteria*, and *Bacteroidetes* as the most dominant phyla in leaves and roots, independent of seed sanitization. This is again consistent with what other studies describe [39]. Relative abundance of *Actinobacteria* and *Bacteroidetes* gradually increased through different developmental stages in mizuna, similar to red Romaine lettuce. Comparing between mizuna mustard leaves and roots, *Firmicutes* were present throughout, and their abundance gradually decreased through the time course. Recently proposed super phylum *Patescibacteria* that encompasses mostly unculturable bacteria from habitats such as groundwater, deep-sea sediments, permafrost, and the continental deep subsurface was also one of the dominant phyla in roots. It has been suggested that members of phylum *Patescibacteria* to have a symbiotic life-style [40, 41].

Invasion by pathogenic microbes often correlates with shifts in microbial communities in different plant compartments, including leaves [42] and roots [43]. Our results showed that, members of the class *Gammaproteobacteria*, such as *Pseudomonas*, *Ralstonia*, *Massilia,* and *Acinetobacter* are some of the dominant genera present in each leafy crop. The same genera have been identified as crucial parts of other plant microbiomes, either as indicators of plant health [44] or their role against invading pathogens [45–47] (Fig. S3). Moreover, in our case, most of these core genera were found to be significantly correlated with *E. coli* abundance, similar to what has been shown for *Fusarium* wilt of banana plants [44], wheat stripe rust [48] and *Rhizoctonia solani* in lettuce plants [45].

We found minimal changes in the diversity of lettuce or mizuna leaf tissue with time or between sanitized and unsanitized seeds, although there were changes in the number of OTUs (Operational Taxonomic Units) with time. Overall, richness was lower in leaf tissue than in root tissue. Studies with model organisms such as *Arabidopsis* have shown similar trends [32]. Additionally, root tissues are more diverse than leaf tissues. Time (age) may also have a significant effect, a factor that we saw influencing diversity between samples. Time had more of an influence on all samples versus the *E. coli* introduction as shown in the beta diversity. Whereas the time course of 7 to 28 days accounted for over 50% of the variation in some samples, the *E. coli* treatment and the combined time-treatment accounted for much less of the diversity.

Bacteria on leaf surfaces are affected by environmental conditions such as temperature and humidity; however, these factors were maintained at steady settings in controlled environments, and therefore should have had minimal impact on the bacterial community. The dynamic changes seen in the phyllosphere could not be explained by environmental factors. In general, there were lower diversities in the phyllosphere, especially at day 7, with diversity increasing with time. At day 7, approximately 10 genera were found in most leaf tissues but by 14–21 days, the number more than doubled with the *E. coli* non-treated samples having more genera than the *E. coli*-treated samples. This could be attributed to genera such as *Bacillus, Ralstonia*, and *Pseudomonas*; however, *Pantoea* and *Massilia* persisted in the leaf tissue from non-treated, unsanitized seeds. *Ralstonia* does not appear to persist in the leaf tissue from *E. coli-*treated, unsanitized seed, however as the *E. coli* declined, *Pseudomonas* and *Burkholderia* both increased, indicating a possible competition for resources. In the leaf tissue from sanitized seeds that are either treated or untreated with *E. coli*, we see *Ralstonia* present and increase with time. In addition, similar to the unsanitized seeds, *Pseudomonas* and *Sphingomonas* both increase in samples from the *E. coli*-treated seed until day 21. *Pseudomonas* parallels *E. coli* and begins to decline whereas *Sphingomonas* continues to increase through day 28. Innerebner et al. [49] have shown that some *Sphingomonas* species have the capacity to outcompete certain *Pseudomonas* species thereby serving as a nutrient-related beneficial microbe.

Root tissue had a higher diversity than leaf tissue as expected as the rhizospheres and roots possess a higher number of genera. Some species were present only in the roots of unsanitized seeds indicating they may be removed from the seed surface during the sanitization process. These include *Hydrotalea*, *Bacillus*, and *Micrococcus*. *Massilia*, a root-colonizing bacterium was present in the roots from unsanitized seeds in high abundance. Ecologically a generalist, it has been found to be present in the early stages of plant development, proliferates on the seed coat and may be sensitive to surrounding media [32, 50]. We saw either a marked decline or absence of *Massilia* in treated samples. This may be due to the competition with *E. coli* for nutrients or due to the media and fertilizers used in this study. Additional studies could provide resolution to this observation. This genus, which can also inhabit the phyllosphere and spermosphere, was only present in the leaves and roots of unsanitized, non-treated seeds indicating that sanitization of the seed had an additional effect on the bacterial community. One additional genus that warrants investigation is *Stenotrophomonas,* which was identified only in the root tissue of sanitized, *E. coli*-treated seeds. Previously classified as a pseudomonad, *Stenotrophomonas* has been isolated in soil, plant, water and human tissues. In soil and plants, it is known for production of plant hormones and is very persistent in the roots, and once established, is not easily out-competed [51, 52].

## Conclusions

Microbial interactions with seeds can influence plant growth and development. Seed surface sanitization is commonly practiced in plant tissue culture and some commercial crop production operations and is a requirement for space crop agriculture for ISS. We investigated the effects of seed surface sanitization on plant-associated microbial communities of two leafy crops, red Romaine lettuce and mizuna mustard with and without introduction of *E. coli* across four time points. Our results showed that *E. coli* persisted for a longer duration on plants from sanitized seeds compared to unsanitized seeds. Although we did not observe any growth and developmental constraints on plants, we did observe dynamic changes in the leaf- and root-associated microbial communities of both crops due to seed surface sanitization. Our results indicated that the seed surface sanitization, a requirement for sending seeds to space, might be altering the plant-associated microbiome, which could potentially influence the plants’ growth and development.

## Methods

### Experimental design

The experimental design for this study was a multi-factorial design. It included two crops, red Romaine lettuce (*Lactuca sativa* cv. Outredgeous) and mizuna mustard, (*Brassica rapa var. japonica*) (Johnny’s Select Seeds, Fairfield, Maine, United States)*.* Effects of seed sanitization (sanitized vs. unsanitized) and *Escherichia coli,* ATCC 11775 (hereon referred as *E. coli*) treatment (treated vs. non-treated) on the microbial community in plant tissue (leaf and root) through four different time-points (7, 14, 21 and 28 days after planting (DAP)).

### Seed sanitization

Crop seeds were sanitized using a bleach/HCl fuming method as described previously [20, 53]. Approximately 75–100 seeds were batch sanitized by adding 0.5 ml concentrated HCl to 30 ml bleach (5% sodium hypochlorite solution) in a 500 ml wide-mouth jar and placing an open petri dish containing the seeds into the bleach container, without submersion, then sealing the container to maintain the chlorine fumes around the seeds. Seeds were sanitized for 1 h before being removed from the acid/bleach container for off-gassing overnight (approximately 24 h) in a laminar flow hood. Seed sanitization was confirmed for both bacterial and fungal contaminants as well as germination rate. To verify effectiveness of sanitization, 20 seeds were placed on 2 plates of Trypticase Soy Agar (TSA) and 2 plates of Inhibitory Mold Agar (ISA) thus 5 seeds per plate. Enumeration of bacteria and fungi was also done by placing 20 seeds in 1 ml sterile phosphate buffered saline (PBS) with sterile glass beads (3 mm) vortexing for 2 min and plating in duplicate 100 μl on TSA and IMA. All plates were incubated at 30 °C for 24–48 h for TSA and up to 5 days for IMA. Plates containing seeds were observed daily for any growth around the seed while colonies were counted at the end of the incubation period from those plated for enumeration. The same analysis was performed on unsanitized controls. CFUs per seed on sanitized seeds were below detection limits (0.5 CFU/seed) for TSA plates. CFUs per unsanitized seed were 0.5 on red Romaine lettuce and 1.0 on mizuna mustard. Germination rate was determined by the number of seeds (out of 10) that germinated on moist filter paper within that same period.

### Seed inoculation

Seeds from both crops were prescreened for the presence of *E. coli* prior to use by 16S rRNA sequencing and determined to be *E. coli* free. Seeds from each of the two crop plants were prepared in sufficient quantities with four treatments: 1) sanitized seeds exposed to *E. coli*; 2) sanitized seeds without exposure to *E. coli*; 3) unsanitized seeds exposed to *E. coli*; and 4) unsanitized seeds without exposure to *E. coli*. A cell culture of *E. coli* was prepared in trypticase soy broth (TSB) and incubated at 30 °C for 16 h in a shaking incubator. Fifty seeds were inoculated by submersion in a 5 ml culture of 10^6^ *E. coli* cells ml^− 1^*,* overnight, at room temperature just prior to planting. Seeds not treated with *E. coli* were submerged in sterile TSB under the same conditions.

### Plant cultivation and harvest

Four seeds were planted in 10-cm (4-in.) square pots containing (~ 500 ml) greens grade, Turface, calcined clay (arcillite) (Ewing Irrigation, St Petersburg, FL, United States) and 7.5 g liter^− 1^ of 18–6-8, type 180-day time-release fertilizer (Nutricote Florikan ESA, Sarasota, FL, United States). Seeds were germinated and grown in controlled-environment growth chambers simulating environmental conditions aboard the ISS (50% relative humidity, 3000-ppm CO_2_, temperature 23 °C). The photoperiod was set at 16 h/8 h light dark cycles with fluorescent lights providing 200–300 μmol m^− 2^ s^− 1^ photosynthetically active radiation (PAR). All plants of the same treatments were contained in a single tray and bottom watered with DI water using an automated watering system and monitored daily. Pots were thinned to a single plant per pot at day 7 and additional harvests were completed every 7 days until day 28. Each harvest included three plants, chosen at random, separated into leaf and root tissue. All harvests were completed using aseptic techniques to minimize contamination. Plant tissues were separated into leaf and root and placed in pre-weighed 50-ml tubes containing sterile water and glass beads.

### Cell count

Plants tissues were placed in a 50 -ml sterile, centrifuge tube with 10–20 ml sterile water and 3-mm glass beads then placed in an OMNI BeadRupter for tissue disruption (OMNI International, Kennesaw, GA, United States). All sample weights were determined prior to disruption. Sample extracts were diluted into PBS and 100 μl of appropriate dilutions were plated in duplicate onto trypticase soy agar (TSA) (BBL, Difco, Becton Dickinson, Franklin Lakes, NJ, United States) and incubated at 30 °C for 48 h before enumeration of colonies. Count data were determined for colony forming units/gram fresh weight (CFU gfw^− 1^) then log transformed.

### DNA isolation, qPCR and 16S PCR with sequencing

The remaining liquid was centrifuged at 13,000 x g and the pellet was processed for DNA isolation using the UltraClean Microbial DNA Isolation Kit per manufacturer’s protocol (Qiagen, Carlsbad, CA, United States). DNA was quantified with the Qubit ds DNA assay (Invitrogen Inc., Grand Island, NY) for downstream qPCR, 16S PCR, and sequencing.

Quantitative PCR (qPCR) was performed on the Roche LightCycler 480 using the SYBR Green Master Kit (Roche Diagnostics, Indianapolis, IN, United States) with 200 nM each of the *ycfR* gene primers unique to *E. coli* (5’TAAGCTCCATGTCATTTGCC-3′ Forward and 5’TTCCATGGAGGGTATTCGG-3′ Reverse) and 5 μl DNA template for a total of up to 2.5 ng of genomic DNA. The *ycfR* gene was selected, as it is present in one copy per cell in this *E. coli* strain and strain specific primers were available. The *ycfR* gene, though not specific to *E. coli*, produces a stress resistant protein, is a biofilm regulator in *E. coli,* and may serve as a surrogate to *E. coli* O157:H7 [54, 55]. A standard curve was generated with DNA isolated from *E. coli* with concentration ranging from 10^0^ to 10^− 7^ ng of genomic DNA. The qPCR cycling conditions included a 10 min denaturation cycle followed by 45 amplification cycles (95 °C for 10 s, 54 °C for 1 min, and 72 °C for 30 s). The melt curve was determined at 95 °C for 5 s, 65 °C for 1 min then 95 °C). All standards and samples were run in triplicate. Copy number was converted to *E. coli* cell number gfw^− 1^ and log transformed.

### 16S PCR and sequencing

The 16S PCR was completed with custom barcoded 16S rRNA gene primers (V4 region) as described by Kozich et al. [56]. The final PCR master mix contained 1X PCR buffer, 2.25 mM MgCl_2_, 300 nM dNTPs, 300 nM forward and reverse primers, and 0.25 units of Platinum Taq polymerase (Invitrogen, Grand Island, NY, United States). Each reaction was completed in duplicate with 1 ng of DNA per reaction. The samples were denatured at 95 °C for 5 min followed by 30 cycles at 95 °C for 1 min, 58 °C for 1 min and 72 °C for 2 min. A final elongation step at 72 °C for 10 min completed the PCR. Replicates of each PCR reaction were combined, and the reaction cleaned with the Min-Elute Cleanup System to remove excess primers and dNTPs (Qiagen, Carlsbad, CA, United States). Amplicon concentration was determined with the Qubit 2.0 broad range ds DNA assay (Invitrogen, Grand Island, NY, United States) and nanomolar concentration calculated for each sample. Samples were diluted to 4 nM and an equimolar concentration library was created per Illumina’s library preparation guidelines. The library was spiked with a 10% PhiX aliquot to add diversity to the library and sequenced on the Illumina MiSeq platform (Illumina Inc., San Diego, CA, United States) with a 500-cycle V2 kit with 250-bp paired ends and FASTQ reads at > 30 quality score.

### Sequencing data analysis

Data generated from the16S rRNA amplicon sequencing of community samples were analyzed using QIIME2 (ver. 2020.2) [57]. Demultiplexed raw sequences were imported into the DADA2 de-noising algorithm to process the raw sequences into exact sequence variants (ESVs). The DADA2 pipeline performed filtering, de-replication, chimera identification and the merging of paired-end reads [58]. The taxonomies were assigned using SILVA 132 rRNA gene database available for use with QIIME2-feature-classifier plugin [59]. Exact sequence variants affiliated with chloroplasts and mitochondria were removed in order to keep only microbial sequences [60, 61]. We used the ‘qiime feature-table filter-features’ to select features that are present in a minimum of six samples (−-p-min-samples 6). All other analyses contained a full data set. A differential abundance analysis was carried out by importing essential QIIME2 artifacts using qiime2R [62] into a phyloseq [63] object, followed by the edgeR [64] analysis to identify taxa that are differentially abundant across the time course.

### Statistical analysis

Differences between plate counts (CFU) and *E. coli* cell number from sanitized and unsanitized treatments in the treated and non-treated mizuna tissues (*P* < 0.05) were determined using a two-way ANOVA followed by Tukey’s multiple comparison test. Data from lettuce were analyzed using a mixed model as implemented in GraphPad Prism 8. In the absence of missing values, this method gives the same *P* values and multiple comparisons tests as repeated measures ANOVA. In the presence of missing values (as in lettuce data set), the results can be interpreted like repeated measures ANOVA. Graphical representation was completed in GraphPad (GraphPad Prism version 8.0.0 for Windows. GraphPad Software, San Diego, CA, www.graphpad.com).

Alpha diversity represented as Shannon Index [65] and beta diversity represented by Bray–Curtis dissimilarity [66] were calculated using the diversity plugin in QIIME2 [57]. Both alpha and beta diversity were calculated using features (in this case OTUs) as frequencies as the default input to the ‘qiime diversity alpha/beta’ command. The Kruskal-Wallis pairwise test was implemented to compare alpha diversity values between each time-point for sanitized and unsanitized samples from leaf and root [67]. A Bray-Curtis dissimilarity matrix was constructed to estimate the global differences in leaf and root samples due to 1) *E.coli* treatment vs. non-treatment, 2) sanitized vs. unsanitized seeds, across all four time-points and visualized via a Principal Coordinate Analysis (PCoA). A Permutational Multivariate Analysis of Variance Using Distance Matrices (adonis) was completed using “qiime diversity adonis”. To assess global differences between treatments and the time-course (DAP), option --p-formula “treatment*time-course” was implemented [68]. *P*-values on the PCoA plots indicate statistical significance of the R^2^ value. Taxa represented in differential abundance plots represent a statistical significance of *P* < 0.05. Heatmaps were created using ampvis2 package in R [69].

## Supplementary Information

**Additional file 1: Supplemental Figure S1.** Boxplots representing alpha diversity in red Romaine lettuce. Top graphs (A-D) represent lettuce leaves while bottom graphs (E-H) represent lettuce root. The sequence of treatment across each row is Sanitized seed, *E. coli* treated (A, E); Sanitized seed, non-treated (B, F); Unsanitized seed, *E. coli* treated (C, G); and Unsanitized seed, non-treated (D, H) plant tissue. Alpha diversity was determined using the QIIME2 package on the 16S rRNA sequencing data. **Supplemental Figure S2.** Boxplots representing alpha diversity in mizuna mustard. (Top graphs (A-D) represent mizuna leaves while bottom graphs (E-H) represent mizuna root. The sequence of treatment across each row is Sanitized seed, *E. coli* treated (A, E); Sanitized seed, non-treated (B, F); Unsanitized seed, *E. coli* treated (C, G); and Unsanitized seed, non-treated (D, H) plant tissue. Alpha diversity was determined using the QIIME2 package on the 16S rRNA sequencing data. **Supplemental Figure S3.** Core microbiomes for red Romaine lettuce and mizuna mustard. (A) Venn diagram showing common genera between sanitized and unsanitized seed generated, leaf and root tissues of red Romaine lettuce. (B) Venn diagram showing common genera between sanitized and unsanitized seed generated, leaf and root tissues of mizuna mustard. Table shows genera represented by the “core microbiome” for red Romaine lettuce (27% from Venn diagram) and mizuna mustard (24.5% from Venn diagram).

## Data Availability

The datasets generated and/or analyzed during the current study will be made available through the NASA GeneLab data repository, an open-access resource. The lettuce data can be accessed via the accession number GLDS-385 and 10.26030/esef-7r30 (10.26030/esef-7r30). The mizuna data can be accessed via the accession number GLDS-386 and 10.26030/thwa-cn80 (10.26030/thwa-cn80).

## Abbreviations

TSA: Trypticase Soy Agar
TSB: Trypticase Soy Broth
APC: Aerobic Plate Count
gfw: gram fresh weight
CFU: Colony-Forming Unit
ISS: International Space Station
NASA: National Aeronautics and Space Administration
QPCR: Quantitative Polymerase Chain Reaction
OTUs: Operational Taxonomic Units
PCoA: Principle Co-ordinate Analysis
ESVs: Exact Sequence Variants
