# Supplementary material for: Persistence of Escherichia coli in the microbiomes of red Romaine lettuce (Lactuca sativa cv. ‘Outredgeous’) and mizuna mustard (Brassica rapa var. japonica) - does seed sanitization matter?
Source: BMC Microbiol. 2021 Oct 22;21:289. doi: 10.1186/s12866-021-02345-5 (PMC8532290; doi:10.1186/s12866-021-02345-5)
Supplement: Supplementary file 1 — Additional file 1: Supplemental Figure S1. Boxplots representing alpha diversity in red Romaine lettuce. Top graphs (A-D) represent lettuce leaves while bottom graphs (E-H) represent lettuce root. The sequence of treatment across each row is Sanitized seed, E. coli treated (A, E); Sanitized seed, non-treated (B, F); Unsanitized seed, E. coli treated (C, G); and Unsanitized seed, non-treated (D, H) plant tissue. Alpha diversity was determined using the QIIME2 package on the 16S rRNA sequencing data. Supplemental Figure S2. Boxplots representing alpha diversity in mizuna mustard. (Top graphs (A-D) represent mizuna leaves while bottom graphs (E-H) represent mizuna root. The sequence of treatment across each row is Sanitized seed, E. coli treated (A, E); Sanitized seed, non-treated (B, F); Unsanitized seed, E. coli treated (C, G); and Unsanitized seed, non-treated (D, H) plant tissue. Alpha diversity was determined using the QIIME2 package on the 16S rRNA sequencing data. Supplemental Figure S3. Core microbiomes for red Romaine lettuce and mizuna mustard. (A) Venn diagram showing common genera between sanitized and unsanitized seed generated, leaf and root tissues of red Romaine lettuce. (B) Venn diagram showing common genera between sanitized and unsanitized seed generated, leaf and root tissues of mizuna mustard. Table shows genera represented by the “core microbiome” for red Romaine lettuce (27% from Venn diagram) and mizuna mustard (24.5% from Venn diagram). [file 12866_2021_2345_MOESM1_ESM.docx]

**A**

**B**

**C**

**D**

**EE**

**F**

**G**

**H**


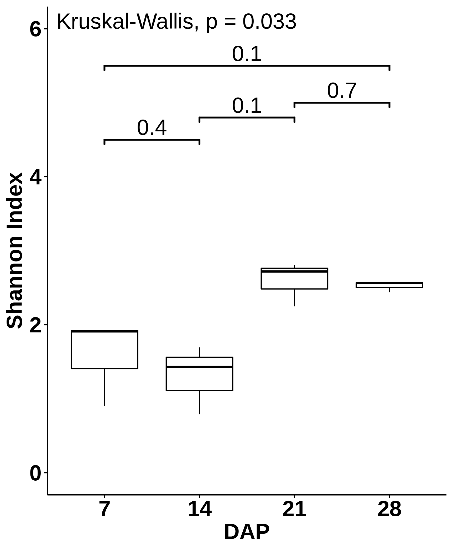

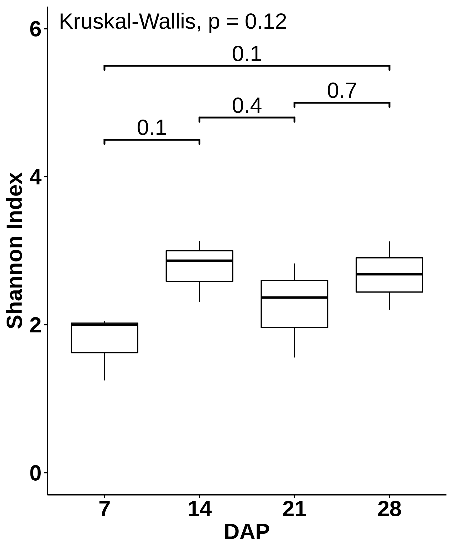

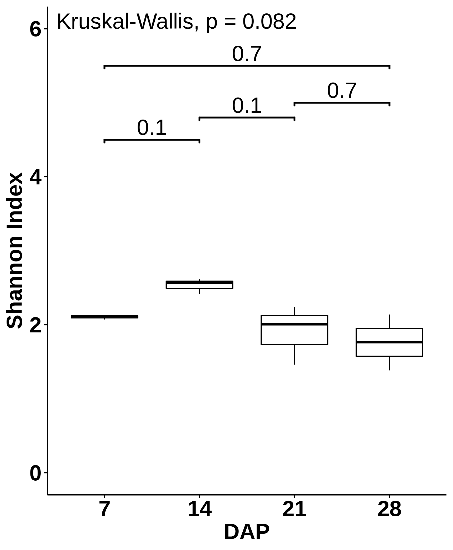

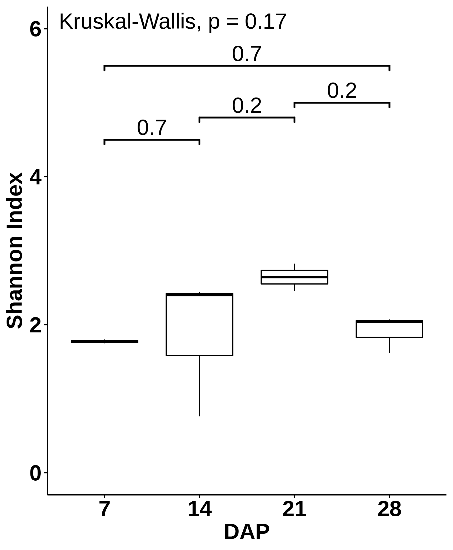

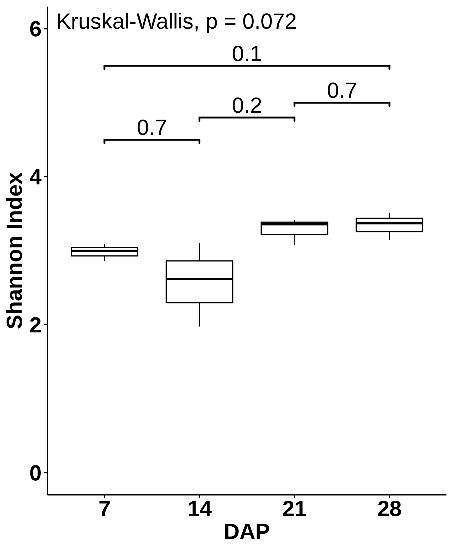

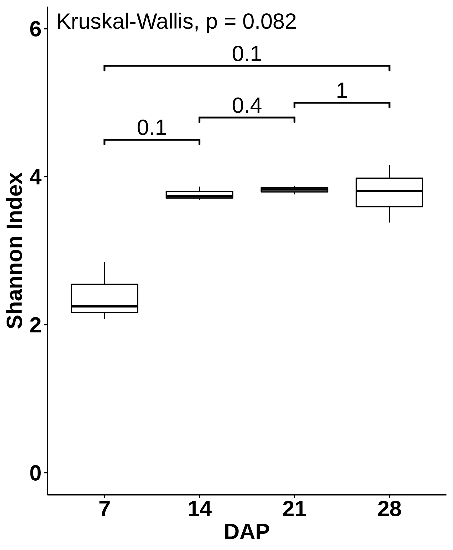

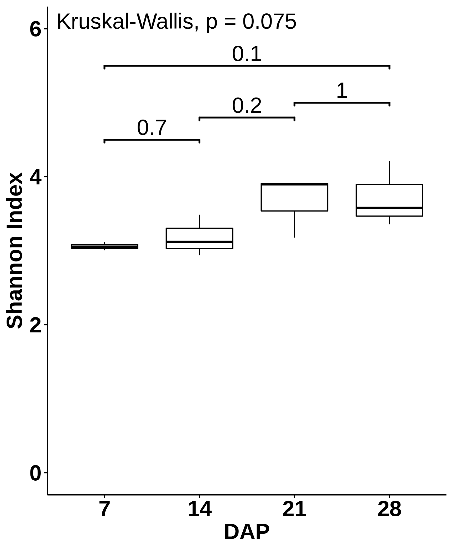

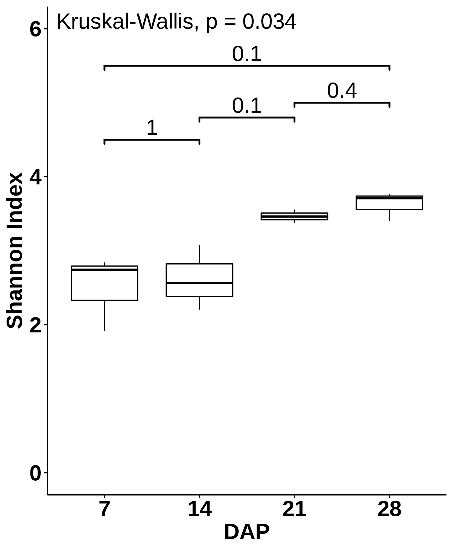


**Supplemental Figure S1**: Boxplots representing alpha diversity in red Romaine lettuce. Top graphs (A-D) represent lettuce leaves while bottom graphs (E-H) represent lettuce root. The sequence of treatment across each row is Sanitized, *E. coli* treated (A, E); Sanitized, non-treated (B, F); Unsanitized, *E. coli* treated (C, G); and Unsanitized, non-treated (D, H) plant tissue. Alpha diversity was determined using the QIIME2 package on the 16S rRNA sequencing data.

**A**

**C**

**D**

**EE**

**F**

**G**

**H**


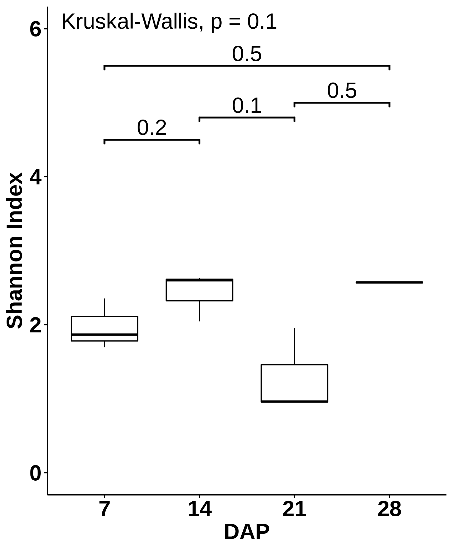

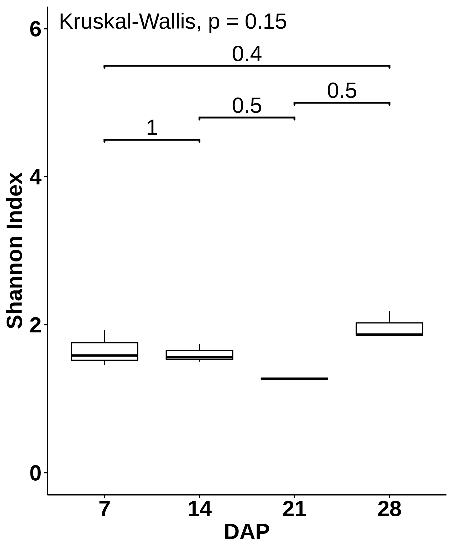

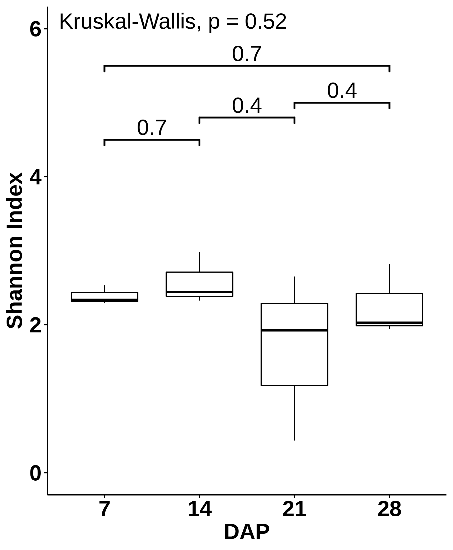

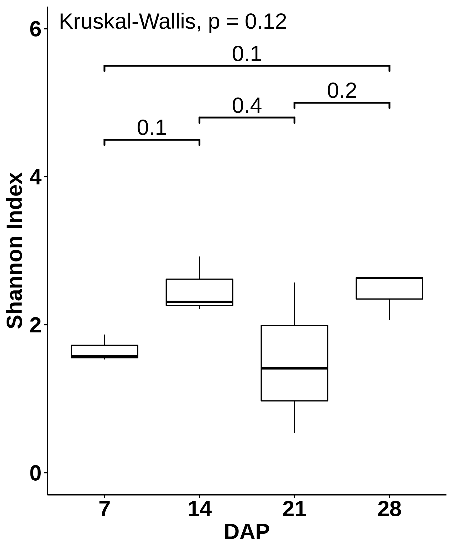

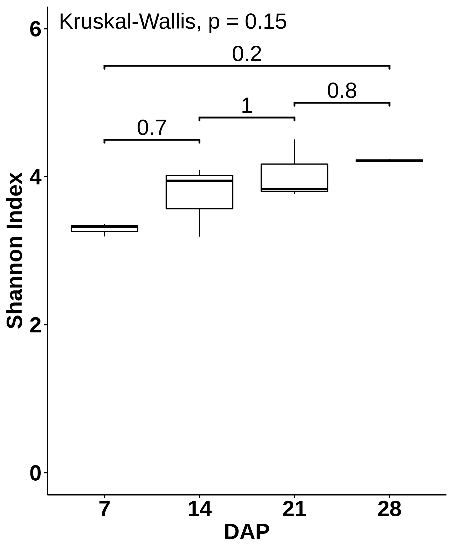

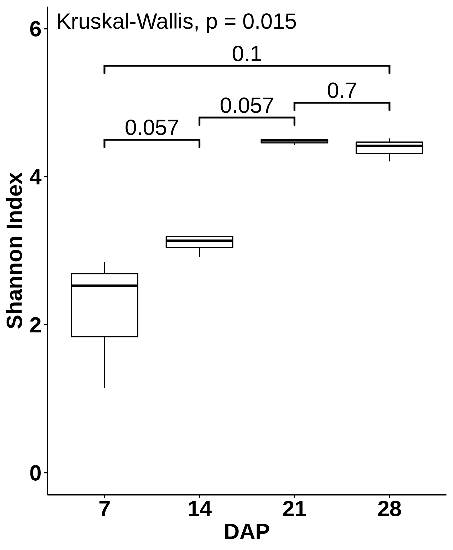

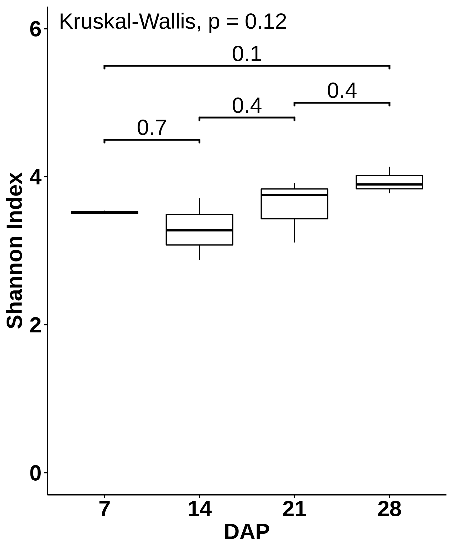

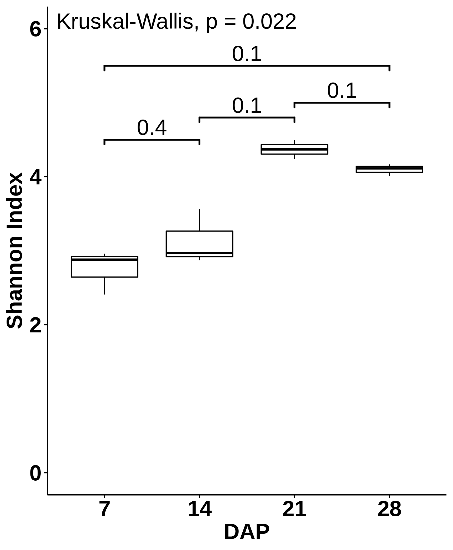


**B**

**Supplemental Figure S2**: Boxplots representing alpha diversity in mizuna mustard. (Top graphs (A-D) represent mizuna leaves while bottom graphs (E-H) represent mizuna root. The sequence of treatment across each row is Sanitized, *E. coli* treated (A, E); Sanitized, non-treated (B, F); Unsanitized, *E. coli* treated (C, G); and Unsanitized, non-treated (D, H) plant tissue. Alpha diversity was determined using the QIIME2 package on the 16S rRNA sequencing data.

| Core Microbiomes | |
| --- | --- |
| Red Romaine Lettuce | **Mizuna Mustard** |
| *Acinetobacter* | *Acinetobacter* |
| *Bradyrhizobium* | *Allorhizobium-Neorhizobium-Pararhizobium-Rhizobium* |
| *Burkholderia-Caballeronia-Paraburkholderia* | *Azospirillum* |
| *Escherichia-Shigella* | *Bacillus* |
| *Massilia* | *Escherichia-Shigella* |
| *Methylophilus* | *Massilia* |
| *Pseudomonas* | *Methylobacterium* |
| *Ralstonia* | *Methylophilus* |
| *Sphingobium* | *Ralstonia* |
| *Sphingomonas* | *Sphingobium* |
|  | *Sphingomonas* |


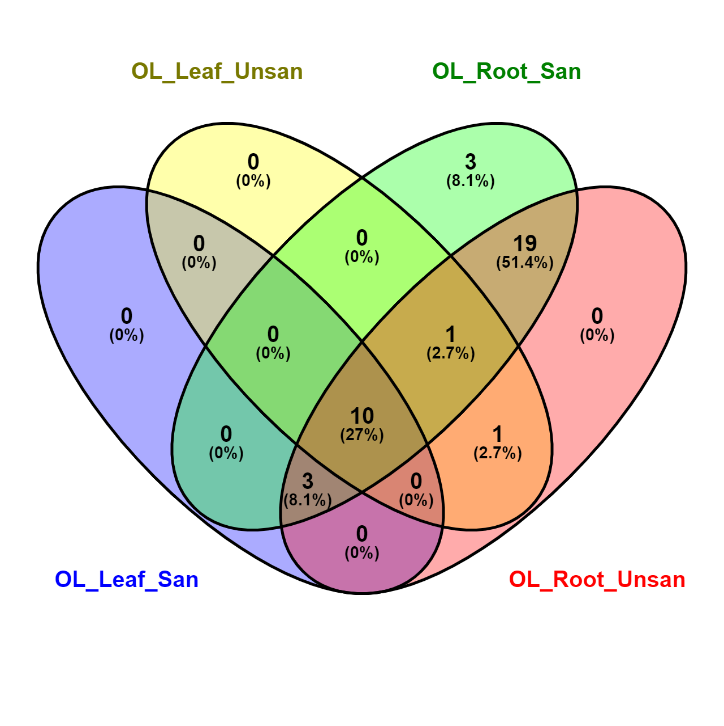


**A**

**B**


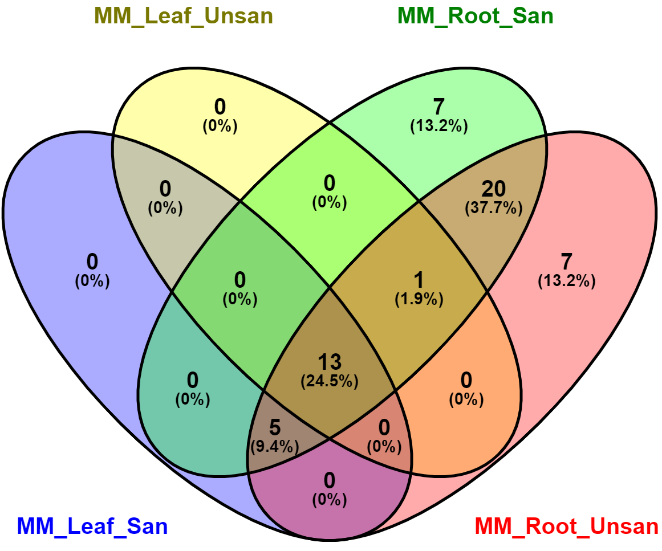


**Supplemental Figure S3:** Core microbiomes for red Romaine lettuce and mizuna mustard. (A) Venn diagram showing common genera between sanitized and unsanitized, leaf and root tissues of read Romaine lettuce. (B) Venn diagram showing common genera between sanitized and unsanitized, leaf and root tissues of mizuna mustard. Table shows genera represented by the “core microbiome” for red Romaine lettuce (27% from Venn diagram) and mizuna mustard (24.5% from Venn diagram).
